# A New Hope in Type 2 Diabetes Mellitus Management: Sodium-Glucose Cotransporter 2 Inhibitors

**DOI:** 10.7759/cureus.18300

**Published:** 2021-09-26

**Authors:** Pallavi Prakash Chaurasia, Sagar Dholariya, Fenilkumar Kotadiya, Milav Bhavsar

**Affiliations:** 1 Internal Medicine, RotaCare Clinic, Walnut Creek, USA; 2 Biochemistry, All India Institute of Medical Sciences, Rajkot, Rajkot, IND; 3 Internal Medicine, Charleston Area Medical Center, Charleston, USA; 4 Biochemistry, C.U. Shah Medical College, Surendranagar, IND

**Keywords:** clinical trials, diabetic kidney disease, cardiovascular disease, dapagliflozin, empagliflozin, canagliflozin, type 2 diabetes mellitus

## Abstract

Diabetes mellitus is a chronic disease that affects multiple organs and exhibits significant complications. The major outcomes of prolonged hyperglycemia are nephropathy, retinopathy, neuropathy, and cardiovascular events due to the glycation of lipids and proteins. To ensure a healthy lifestyle for diabetic patients, a treatment that delays the complications and simultaneously protects multiple organs is required. Sodium-glucose cotransporter inhibitors (SGLTi) inhibit the reabsorption of glucose from the kidney and shows promising benefits in renal and heart diseases. The major SGLT receptors are SGLT1 and SGLT2. Various trials are conducted to conclude their efficacy and show nephroprotective and cardioprotective roles independent of diabetic status. The FDA-approved SGLT2 inhibitors are empagliflozin (Jardiance®), canagliflozin (Invokana®), and dapagliflozin (Farxiga®), which are primarily used in type 2 diabetes mellitus (T2DM). They show a reduced rate of hospitalization for heart failure, cardiovascular disease mortality, all-cause mortality, and progression of diabetic kidney disease. It also shows improvement in the glycemic index; therefore, it is protective against the complications of diabetes irrespective of insulin release, thus avoids hypoglycemia. This review summarizes the data from the clinical trials that support the efficacy of SGLT2 inhibitors in reducing the risks of cardiovascular and renal outcomes in patients with T2DM.

## Introduction and background

Type 2 diabetes mellitus (T2DM) is one of the most prevalent diseases. It is now estimated that nearly 387 million people have diabetes globally, and deaths from diabetes are projected to increase 50% worldwide by 2025 [1]. The sedentary lifestyle and unhealthy eating habits are contributing to the increased prevalence of diabetes mellitus. T2DM is a multifactorial progressive disease and is caused by genetic, environmental, dietary, metabolic, and sedentary lifestyles. Management of diabetes mellitus requires a drug with high efficiency and a lower risk of hypoglycemia.

Sodium-glucose cotransporter-2 (SGLT2) inhibitors are a new class of hypoglycemic drugs with promising health benefits. These drugs inhibit the reabsorption of glucose from the kidney; thus, they increase glycosuria, improve the glycemic index without affecting insulin release, and avoid hypoglycemia. SGLT receptors present in the luminal surface of epithelial cells are of low capacity; high-affinity glucose-sodium cotransporters reabsorb the filtered glucose against a concentration gradient [1-3]. The most important SGLT receptors are SGLT1 and SGLT2 receptors. Type 1 receptors are present in the small intestine and late proximal convoluted tubules and are responsible for the reabsorption of 10% of filtered glucose, while SGLT2 receptors are responsible for 90% glucose reabsorption from early proximal convoluted tubules, the main target of antidiabetic therapy [4-6]. The renal threshold for glucose is 180 mg/dl in the normoglycemic state, but in diabetes, it may reach up to 200-240 mg/dl and may exacerbate hyperglycemia. Studies show the increased expression of SGLT2 receptors, SGLT1 proteins, and mRNA in diabetic kidneys, thus increasing the reabsorption of glucose [7-11]. The US Food and Drug Administration (FDA) first approved the use of SGLT2 inhibitors in 2013 for patients with T2DM, and the first study was published in 2016, which demonstrated their beneficial effects in terms of delaying diabetic kidney disease (DKD) progression [12]. The class of SGLT2 inhibitors approved by the FDA in T2DM includes canagliflozin (Invokana®), dapagliflozin (Farxiga®), and empagliflozin (Jardiance®) [13].

**Figure 1:**
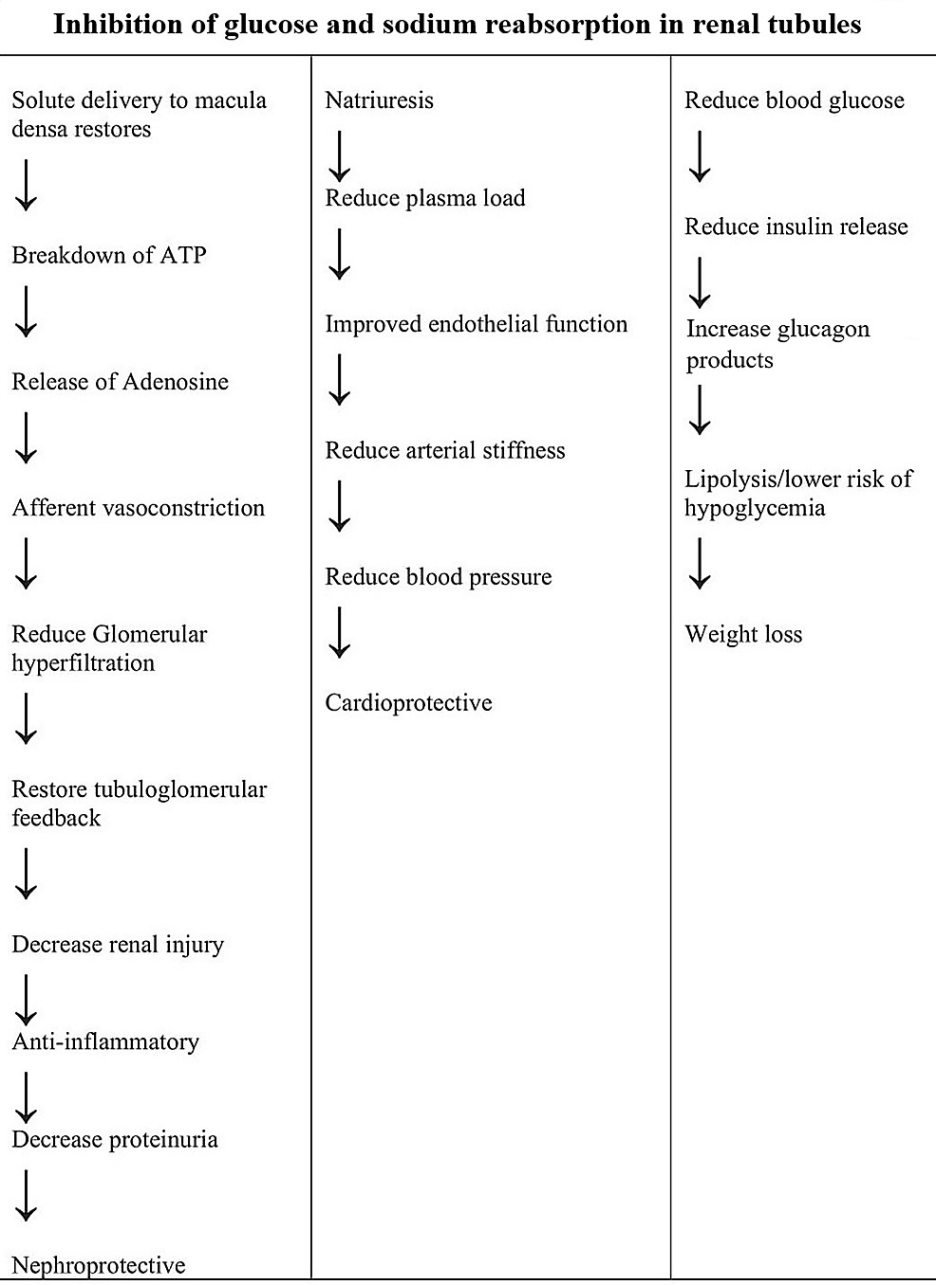
Mechanism of SGLT2 inhibitors ATP, Adenosine triphosphate; SGLT, sodium-glucose cotransporter.

In this review, we summarize the cardiac and renal benefits of SGLT2 inhibitors in T2DM based on the results of major randomized controlled trials and previously conducted studies. Figure 1 demonstrates various mechanisms responsible for the beneficial effects of SGLT2 inhibitors.

**Figure 2:**
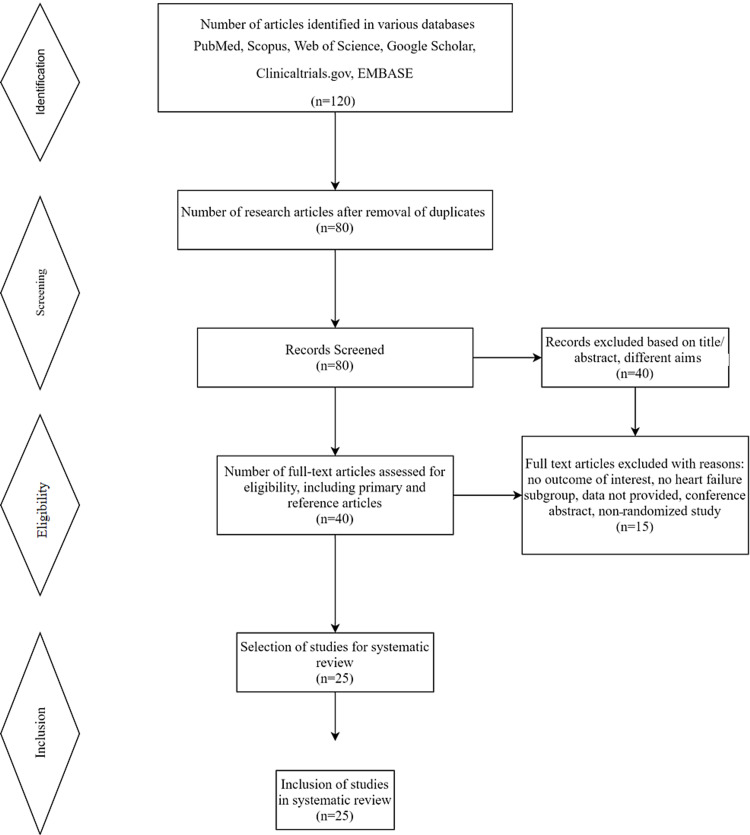
PRISMA guidelines for screening and selection of studies PRISMA, Preferred Reporting Items for Systematic Reviews and Meta-Analyses.

We systematically searched PubMed, Scopus, Web of Science, Google Scholar, ClinicalTrials.gov, and other databases for eligible articles using appropriate search words. We extracted data from placebo-controlled randomized controlled trials, original articles, meta-analyses, and systematic reviews that reported cardiovascular, renal, and other positive outcomes of SGLT2 inhibitors in individuals with T2DM. The screening of the articles is carried out by abstract screening and full-text reading to confirm the eligibility. The full text of references within the primary studies was also reviewed and included in the final study as needed. Out of 120 searched articles, a total of 25 articles were included in the final review after removing duplicates of studies, non-randomized trials, studies measuring other perspectives, and studies showing no outcome of interest. The detailed search strategy of the database is shown in Figure 2. The endpoint of the study was the composite cardiovascular (CV) outcomes, CV mortality, all-cause mortality, nonfatal myocardial infarction, nonfatal stroke, and hospitalization for heart failure. The renal composite outcome, albuminuria, and effects of estimated glomerular filtration rate (eGFR) were also studied.

## Review

The major trials including empagliflozin and canagliflozin are Empagliflozin Cardiovascular Outcome Event Trial-Removing Excess Glucose (EMPA-REG OUTCOME) and Canagliflozin Cardiovascular Assessment Study (CANVAS) program, respectively, in T2DM patients. These trials show improvement in cardiovascular disease (CVD) and renal outcomes among 17,000 participants with T2DM [12,14-17]. The participants were randomly allocated to the placebo or empagliflozin group. The empagliflozin group demonstrated remarkable lower incidents of hospitalization for heart failure (35% relative risk reduction), CVD mortality (38% relative risk reduction), and death from any cause (32% relative risk reduction) in contrast to the placebo group [14]. The reduced rates were sustained among more than 2000 participants with an eGFR of less than 60 mL/min/1.73 m^2^ and macroalbuminuria [15,18]. As compared to placebo, there is a 39% relative risk reduction in the new onset or worsening nephropathy in the empagliflozin group (13% vs. 19%, P < 0.001) [12,18].

The CANVAS program consolidated data from two CVD outcome trials, which included 10,000 participants with T2DM, allocated to either canagliflozin or placebo groups, and followed for an average of 3.6 years [17]. In comparison to the placebo, the primary composite outcome occurred in lower rates among canagliflozin takers in terms of CVD mortality, nonfatal myocardial infarction, and nonfatal stroke (14% relative risk reduction, P < 0.001). There is a 27% reduction in progression to albuminuria as well as 40% alleviation in the composite kidney disease outcomes that include 40% eGFR decline, kidney replacement therapy (KRT), or death from renal disease among the canagliflozin group against placebo [17]. The beneficial outcomes were persistent across the various stages of kidney function (eGFR: 30-45, 45-60, 60-90, and >90 mL/min/1.73 m^2^) though canagliflozin showed greater benefits on fatal/nonfatal strokes in eGFR < 60 mL/min/1.73 m^2^ (hazard ratio: 0.56 in the 45-60 mL/min/1.73 m^2^ group and 0.32 in the 30-45 mL/min/1.73 m^2^ group vs. placebo) [19]. Other clinical trials were Canagliflozin and Renal Endpoints in Diabetes With Established Nephropathy Clinical Evaluation (CREDENCE) and Dapagliflozin and Prevention of Adverse Outcomes in Chronic Kidney Disease (DAPA-CKD). These trials were designed to evaluate the effectiveness of canagliflozin or dapagliflozin on composite primary outcomes, which includes end-stage renal disease (ESKD), doubling of serum creatinine, 50% sustained decline in eGFR, and renal or CVD mortality in participants diagnosed with diabetic kidney disease (DKD) [20]. A total of 4401 participants were followed for a mean duration of 2.62 years in the CREDENCE trial. The end result of the CREDENCE trial illustrated a 30% reduction in the relative risk of the primary outcome with the use of canagliflozin as compared to the placebo group, with incident rates of 43.2 and 61.2 per 1000 patient-years, respectively (hazard ratio: 0.70; 95% confidence interval (CI): 0.59-0.82; P = 0.00001). Also, there was a 34% reduction in the relative risk (hazard ratio: 0.66; 95% CI: 0.53-0.81; P < 0.001) of the renal-specific outcome of ESKD, a doubling of the creatinine level, or renal-related mortality. The reduction in the relative risk of ESKD by 32% (hazard ratio, 0.68; 95% CI, 0.54-0.86; P = 0.002) was also noted. The use of canagliflozin also revealed a risk reduction in cardiovascular death, myocardial infarction, or stroke (hazard ratio: 0.80; 95% CI: 0.67-0.95; P = 0.01) and hospitalization for heart failure (hazard ratio: 0.61; 95% CI: 0.47-0.80; P < 0.001) [21].

Another trial evaluated the prevention of adverse outcomes in CKD patients taking dapagliflozin (eGFR 25-75 mL/min/1.73 m^2^ and Urine Albumin Creatinine Ratio [UACR] > 200 mg/g) and in those with and without T2DM [22]. The primary outcome includes a composite kidney outcome (>50% sustained decline in eGFR, ESKD) or renal or cardiovascular mortality. As it demonstrated benefits in the dapagliflozin group, the trial was terminated early; however, full results have not been released [23]. In the Dapagliflozin and Prevention of Adverse Outcomes in Heart Failure (DAPA-HF) trial, dapagliflozin showed a reduction in cardiovascular deaths by 26% along with benefits in heart failure (hospitalization or an urgent visit resulting in intravenous therapy for heart failure). This study evaluates the benefits of SGLT2i in patients with ejection fraction < 40% with or without T2DM [24]. The results of clinical trials are summarized in Table 1.

**Table 1:** Primary and secondary outcomes of clinical trials MACE, Major adverse cardiovascular events; HF, heart failure; HR, hazard ratio; CI, confidence interval; CV, cardiovascular; CKD, chronic kidney disease; ESKD, end-stage kidney disease (eGFR < 15 mL/m/1.73 m^2^); CANVAS, Canagliflozin Cardiovascular Assessment Study; DAPA, dapagliflozin; CREDENCE, Canagliflozin and Renal Events in Diabetes and Nephropathy Clinical Evaluation; Empa-REG Outcome, Empagliflozin Removing Excess Glucose (EMPA-REG) outcome; HHF, hospitalization for heart failure.

Clinical Trials	Drug	Primary Outcome	Secondary Outcome		
			All-Cause Mortality	Hospitalization for Heart Failure	CV Mortality
Empa-REG Outcome [14] (n = 7020)	Empagliflozin	MACE: HR 0.86; 95% CI: 0.74-0.99	HR 0.68; 95% CI: 0.57-0.82	HR 0.65; 95% CI: 0.50–0.85	HR 0.62; 95% CI: 0.49–0.77
CANVAS [17] (n = 10,142)	Canagliflozin	MACE: HR 0.86; 95% CI: 0.75-0.97	HR 0.87; 95% CI: 0.74–1.01	HR 0.67; 95% CI: 0.52–0.87	HR 0.87; 95% CI: 0.72–1.06
CREDENCE [21] (n = 4401)	Canagliflozin	Doubling of creatinine: HR 0.60; 95% CI: 0.48-0.76	HR 0.83; 95% CI: 0.68–1.02	HR 0.61; 95% CI: 0.47–0.80	HR 0.80; 95% CI: 0.67–0.95
		ESKD: HR 0.68; 95% CI: 0.54-0.86			
		CV mortality: HR 0.78; 95% CI: 0.61-1.00			
DAPA-CKD [42] (n = 4304)	Dapagliflozin	Decline of ≥50% in eGFR: HR 0.53; 95% CI: 0.42–0.67	HR 0.69; 95% CI: 0.53–0.88	Nephropathy: HR 0.56; 95% CI: 0.45–0.68	HR 0.71; 95% CI: 0.55–0.92
		New ESKD: HR 0.64; 95% CI: 0.50–0.82			
		CV mortality: HR 0.81; 95% CI: 0.58–1.12			
DAPA-HF [24] (n = 4744)	Dapagliflozin	Worsening HF: HR 0.74; 95% CI: 0.65-0.85	HR 0.83; 95% CI: 0.71–0.97	Nephropathy: HR 0.71; 95% CI: 0.44–1.16	HR 0.75; 95% CI: 0.65–0.85
		HHF: HR 0.70; 95% CI: 0.59-0.83			
		CV mortality: HR 0.82; 95% CI: 0.69-0.98			

The nephroprotective role of these drugs is explained by various mechanisms. The most important is the opposition of glomerular hyperfiltration, which prevents glomerular capillary hypertension, thus renal injury [25]. Increased urinary glucose load causes increased expression of SGLT2 receptors, thus increased reabsorption of glucose and sodium into the tubules, which leads to decreased concentration of sodium reaching macula densa due to which there is a reduction in ATP breakdown and production of adenosine. Adenosine is a strong vasoconstrictor, but its deficiency leads to vasodilation of afferent arterioles, thus hyperfiltration. The altered tubuloglomerular feedback system is responsible for aggravated renal injury [26]. With the use of SGLT2 inhibitors, there is increased sodium carried to macula densa, resulting in increased adenosine production and afferent vasoconstriction, thus a reduction in renal plasma flow and glomerular filtration rate (GFR) and restoration of the tubule-glomerular feedback.

A study explored the effect of empagliflozin in T1D and glomerular hyperfiltration as a decrease in measured GFR (inulin clearance) by 33 mL/min/1.73 m^2^ (172+ 23 mL/min/1.73 m^2^ to 139 + 25 mL/min/1.73 m^2^) in conjunction with decreased plasma flows to the kidney and increased kidney vascular resistance. This effect was only observed in patients with diabetes with glomerular hyperfiltration and not in normal glomerular pressure [27]. Some studies also showed a decrease in the hyperglycemia-mediated generation of reactive oxygen species, thus having additional protective anti-inflammatory and antifibrotic effects [28,29]. Clinical trials show reductions in HbA1c with empagliflozin and dapagliflozin in patients with eGFR > 45 and < 60 mL/min/1.73 m^2^ but not in patients with eGFR < 40 mL/min/1.73m^2^ [30,31]. The analysis of the phase III empagliflozin trials and the CANVAS program also showed the beneficiary effects of weight loss in diabetic patients that were maintained with eGFR as low as 30 mL/min/1.73 m^2^ [19,31]. Low fat may decrease albuminuria and glomerular hyperfiltration, which indirectly protects the diabetic kidney and further supports the renoprotective role of SGLT2 inhibitors [30,32]. Antihypertensive effects of SGLT2 inhibitors are believed to be attributed to natriuresis, weight loss, improved endothelial function, and vascular compliance [33-37]. Empagliflozin, dapagliflozin, and canagliflozin lower the systolic blood pressure by approximately 5 mmHg and diastolic blood pressure by approximately 2 mmHg while maintaining the magnitude of blood pressure reduction depending on eGFR [18,38-42].

**Figure 3:**
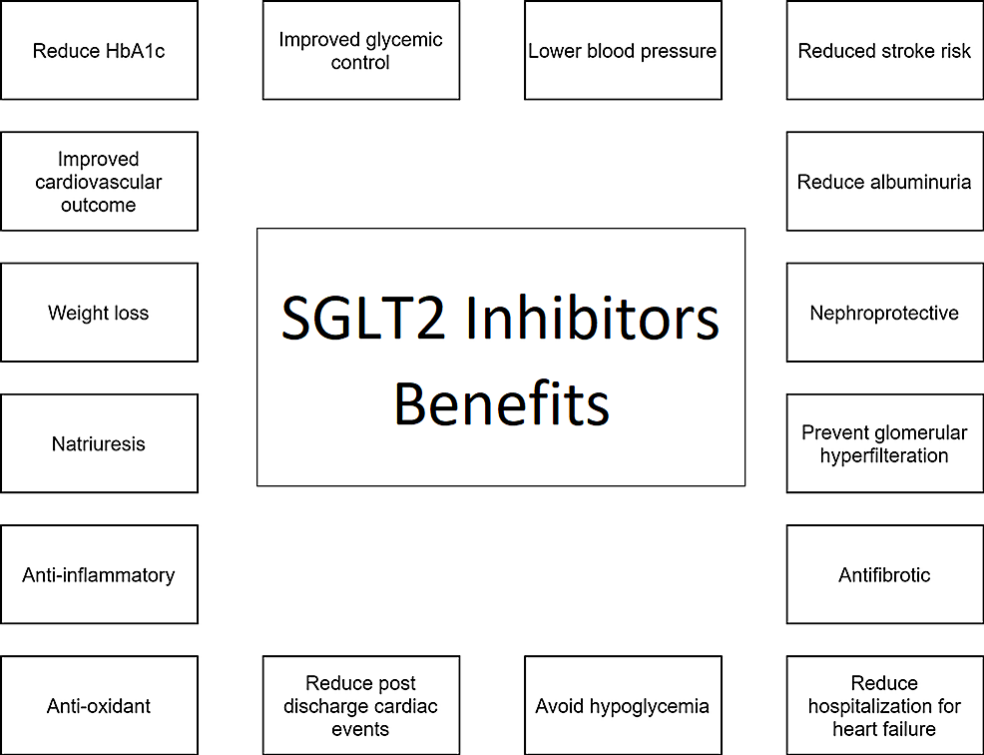
Benefits of SGLT2 inhibitors HbA1c, Glycated hemoglobin; SGLT, sodium-glucose cotransporter.

The summary of the benefits of SGLT2 inhibitors of different body functions is demonstrated in Figure 3.

## Conclusions

The SGLT2 inhibitors appear to play a promising role in preventing cardiac and renal disease and reducing overall mortality in diabetes patients. In addition, their use causes better glycemic control, a decrease in HbA1c levels, and a low risk of hypoglycemia. Furthermore, they decrease the relative risk of stroke, nonfatal myocardial infarction, and hypertension. More benefits include weight loss, delayed albuminuria, natriuresis, anti-inflammatory, and antioxidant properties. The clinical trials demonstrated the drug's potential as a critical diabetes mellitus treatment and in helping to counter off its complications.
